# Supplementary material for: Influence of frailty in older patients undergoing emergency laparotomy: a UK-based observational study
Source: BMJ Open. 2017 Oct 6;7(10):e017928. doi: 10.1136/bmjopen-2017-017928 (PMC5640048; doi:10.1136/bmjopen-2017-017928)
Supplement: Supplementary data [file bmjopen-2017-017928supp001.pdf]

Appendix A: Case Report Form

Q1	Study ID	
Q2	Age at admission to study (years)	
Q3	Sex	Male Female
Q4	Comorbidities	CCF Y/N COPD Y/N<br>CVA Y/N Dementia Y/N<br>Hemiplegia Y/N CKD Y/N<br>Leukaemia Y/N DM(complicated) Y/N<br>Lymphoma Y/N DM(uncomplicated) Y/N Mild liver disease Y/N IHD Y/N<br>Severe liver disease Y/N PVD Y/N<br>Solid tumour Y/N Metastatic tumour Y/N<br>AIDS Y/N Peptic ulcer disease Y/N<br>Connective tissue disease Y/N<br>Other:________________
Q5	Polypharmacy (≥5 medications)	Yes No
Q6	Care level prior to admission*<br><br>**The term "carer" to include both formal and informal care arrangements i.e. friends/*	Home (No carers*)<br>Home (with carers* ____ times/day)<br>Residential Home<br>Nursing home

	relatives	Intermediate care<br><br>Other: ____________________
Q7	Frailty score	
Q8	Interval between admission & surgery (days)	
Q9a	Pre-operative ASA grade	
Q9b	Pre-operative PPOSSUM	Morbidity: Mortality:
Q10a	Operative indication	Peritonitis Perforation<br><br>Abdominal abscess Anastomotic leak<br><br>Intestinal fistula Sepsis (other)<br><br>Intestinal obstruction Haemorrhage<br><br>Ischaemia Colitis<br><br>Abdominal wound dehiscence<br><br>Abdominal compartment syndrome<br><br>Planned relook<br><br>Other ____________________
Q10b	Procedure (circle **ALL** that apply)	Peptic ulcer (suture or repair of perforation)<br><br>Peptic ulcer (oversew of bleeding)<br><br>Gastric surgery - other<br><br>Small bowel resection<br><br>Colectomy: Left (including anterior resection)<br><br>Right Subtotal<br><br>Hartmann's procedure

		Colorectal resection – other<br>Haemostasis Enterotomy<br>Stoma formation Stoma revision<br>Adhesiolysis Intestinal bypass<br>Reduction of volvulus Washout only<br>Abdominal wall closure<br>Drainage of abscess/collection<br>Laparostomy formation<br>Repair of intestinal perforation<br>Resection of other intra-abdominal tumours<br>Exploratory/ re-look laparotomy only<br>Not amenable to surgery<br>Other ____________
Q10c	Primary procedure type	Open<br>Laparoscopic<br>Laparoscopic converted to open<br>Laparoscopic-assisted
Q11a	Length of stay (days)	
Q11b	Readmission to hospital within 30 days	Yes No
Q11c	Reason for readmission	
Q12a	Post-operative complication within 30 days	Yes No
Q12b	Grade of complication	

Q13	Care level on discharge<br><br>**The term "carer" to include both formal and informal care arrangements i.e. friends/ relatives*	Home (No carers*)<br><br>Home (with carers* ____ times/day)<br><br>Residential Home<br><br>Nursing home<br><br>Other: ____________________________
Q14	90 day mortality	Yes No
Q15	Length of ICU/HDU stay	ICU HDU<br><br>Days total stay __________
Q16	Intermediate care stay (days)	Yes No<br><br>Days total stay __________

Appendix B: **Canadian Study of Health and Ageing (CSHA) Frailty Score (Rockwood Score)**

**The CSHA Frailty Scale**	
**1 – Very fit**	Robust, active, energetic, well-motivated and fit; these people commonly exercise regularly and are in the most fit group for their age.
**2 – Well**	Without active disease, but less fit than people in category 1.
**3 – Well, with treated comorbid disease**	Disease symptoms are well controlled compared with those in category 4.
**4 – Apparently vulnerable**	Although not frankly dependent, these people commonly complain of being "slowed up" or have disease symptoms.
**5 – Mildly frail**	With limited dependence on others for instrumental* activities of daily living.
**6 – Moderately frail**	Help is needed with both instrumental* and non-instrumental activities of daily living.
**7 – Severely frail**	Completely dependent on others for activities of daily living, or terminally ill.

- *Non-instrumental activities of daily living are basic everyday tasks such as walking, bathing, dressing, toileting, brushing teeth and eating.*

Instrumental activities of daily living are further tasks such as cooking, shopping, driving etc. Further explanation is available at the following link if required:

https://asourparentsage.net/2009/12/17/adls-and-iadls-whats-the-difference/

Appendix C:

Clavien-Dindo Classification of Surgical Complications

Clavien-Dindo Classification of Surgical Complications	
**Grade I**	Any deviation from the normal postoperative course without the need for pharmacological treatment or surgical, endoscopic, and radiological interventions. Allowed therapeutic regimens are: drugs such as antiemetics, antipyretics, analgetics, diuretics, electrolytes, and physiotherapy. This grade also includes wound infections opened at the bedside.
**Grade II**	Requiring pharmacological treatment with drugs other than allowed for grade I complications. Blood transfusions and total parenteral nutrition are also included.
**Grade IIIa**	Surgical, endoscopic, or radiological intervention that is not under general anesthesia
**Grade IIIb**	Surgical, endoscopic, or radiological intervention that is under general anesthesia
**Grade IVa**	Life-threatening complication requiring intermediate care or intensive care unit management, single organ dysfunction (including dialysis, brain hemorrhage, ischemic stroke, and subarrachnoidal bleeding)
**Grade IVb**	Life-threatening complication requiring intermediate care or intensive care unit management, multi-organ dysfunction (including dialysis)
**Grade V**	Death of a patient
**Suffix "d"**	If the patient suffers from a complication at the time of discharge, the suffix "d" (for "disability") is added to the respective grade of complication. This label indicates the need for a follow-up to fully evaluate the complication

Appendix D: Definitions

Day of study – this is defined by the number of 24h periods passed from first attendance at hospital, the event being examined (e.g. date of operation). Patients admitted and the event occurs with the first 24hrs are classed as 1 day.

Q1. Combination of centre Number and record number. For example if you are in centre 024 and recording data on the 35th patient, the number would be 024.35

Q2. Age in completed years on date of admission to hospital

Q3. Please indicate sex of patient

Q4. These are comorbidities as defined by the Charlson Comorbidity Index. Each should be marked as present if there is any previous documented history of each diagnosis.

**Myocardial infarct**	History of medically documented myocardial infarction
**Congestive heart failure**	Symptomatic congestive heart failure w/ response to specific treatment
**Peripheral vascular disease**	Intermittent claudication, peripheral arterial bypass for insufficiency, gangrene, acute arterial insufficiency, untreated aneurysm (≥6cm)
**Cerebrovascular disease (except hemiplegia)**	History of TIA, or CVA with no or minor sequelae
**Dementia**	Chronic cognitive deficit
**Chronic pulmonary disease**	Symptomatic dyspnoea due to chronic respiratory conditions (inc. asthma)
**Connective tissue**	SLE, polymyositis, polymyalgia rheumatic, moderate to severe

**disease**	rheumatoid arthritis
**Peptic ulcer disease**	Patients who have required treatment for peptic ulcer disease
**Mild liver disease**	Cirrhosis without portal hypertension, chronic hepatitis
**Diabetes (without complication)**	Diabetes with medication
**Diabetes with end organ damage**	Retinopathy, neuropathy, nephropathy
**Hemiplegia (or paraplegia)**	Hemiplegia or paraplegia
**Moderate of severe renal disease**	Creatinine>265umol/L, dialysis, transplantation, uraemic syndrome
**Solid tumour (non-metastatic)**	Initially treated in the last 5 years exclude non-melanomatous skin cancers and in situ cervical carcinoma
**Leukaemia**	CML, CLL, AML, ALL, PV
**Lymphoma, Multiple myeloma**	Non-Hodgkin's Lymphoma, Hodgkin's, Waldenstrȍm, multiple myeloma
**Moderate or severe liver disease**	Cirrhosis with portal hypertension +/- variceal bleeding
**Metastatic solid tumour**	Metastatic solid tumour
**AIDS**	AIDS & AIDS-related complex

Q5. Polypharmacy counted as five or more prescribed regular medications on admission. This includes regular eye drops, inhalers and analgesia.

Q6. Care level prior to admission. Classed as level of social care input prior to admission. Please indicate only one. If patient at own home with daily care input please indicate the number of times each day carers attend.

Q7. Frailty score, 1-7 using the modified Rockwood Scale (please see Appendix B)

Q8. Interval between admission and emergency procedure. Classed as whole days, rounded up to the nearest whole day. (e.g. 0-24h classed as 1 day, 24-48h classed as 2 days)

Q9a. American Society of Anaesthesiologist (ASA) grade:

Grade	Description
1	Healthy individual with no systemic disease
2	Mild systemic disease not limiting activity
3	Severe systemic disease that limits activity but is not incapacitating
4	Incapacitating systemic disease which is constantly life-threatening
5	Moribund, not expected to survive 24 hours with or without surgery

Q9b. P-POSSUM Score: Calculated from pre-morbid status using multiple markers of baseline function including age, cardiac status, observations and blood test results. This should already be routinely documented in all patient notes as part of the National Emergency Laparotomy Audit dataset. If required please use the calculator found at http://www.riskprediction.org.uk/index-pp.php

Q10a. Primary operative indication as per National Emergency Laparotomy Audit data collection

Q10b. Primary operative procedure as per National Emergency Laparotomy Audit collection tool

Q10c. Primary surgical method used during the procedure. NOTE: Laparoscopic assisted should be used if decision to proceed to open was part of the pre-operative procedure planning, otherwise laparoscopic converted to open should be used.

Q11a. Total length of stay of primary admission is defined as number of 24h periods or part thereof, passed from first attendance at hospital, to discharge. Patients admitted and discharged with the first 24hrs are not included in the study, between 24-48hrs 2 day etc.

Q11b. Readmission to hospital within 30 days as an <u>emergency</u> (classed as whole days, rounded up to the nearest whole day) regardless of cause.

Q11c. Reason for readmission to hospital

Q12a. Post-operative complications include:

**Abdominal wall dehiscence**	Full thickness dehiscence of laparotomy wound within 30 days of discharge

**Anastomotic leakage**	A clinical diagnosis will require symptoms related to leakage (gas, pus, or faecal discharge from the drain site, peritonitis or discharge of pus from the rectum). In the event of a clinically suspicious leak (fever or abdominal pain) the diagnosis can be established by operative or radiological diagnosis. When an anastomosis is defunctioned the presence or absence of a leak will be established by contrast radiology.
**Urinary tract infection**	Patient needs to meet two of the following criteria:<br>• Fever $>38^{0}$C<br>• Suprapubic tenderness<br>• Costovertebral angle pain or tenderness<br>• Urinary urgency<br>• Urinary frequency<br>• Dysuria<br>• Urine culture with no more than two species of organisms identified, at least one of which is a bacterium of ≥105 CFU/mL
**Pneumonia**	Patient must meet one of the following criteria:<br>• Dullness to percussion on physical examination of chest and any of the following:<br>- New onset of purulent sputum or change in character of sputum<br>- Organism isolated from blood culture<br>- Isolation of pathogen from specimen obtained from transtracheal aspirate, bronchial brushing or biopsy

	• Chest radiographic examination shows new or progressive infiltrate, consolidation, cavitation or pleural effusion and any of the following<br>- New onset of purulent sputum or change in character of sputum<br>- Organism isolated from blood culture<br>- Isolation of pathogen from specimen obtained from transtracheal aspirate, bronchial brushing or biopsy<br>- Isolation of virus or detection of viral antigen in respiratory secretions<br>- Diagnostic single antibody titre (IgM) or four-fold increase in paired serum samples (IgG) for pathogen
**Superficial SSI**	Patient must meet one of these criteria<br>• Purulent drainage from the incision<br>• At least two of – pain, localised swelling, redness, head, fever AND the incision is opened deliberately to manage infection or the clinician diagnoses a SSI<br>• Wound organisms AND pus cells from aspirate/swab
**Deep (intra-abdominal) SSI**	Patient must meet one of these criteria<br>• A clinical diagnosis of wound infection with dehiscence of mass closure or any layer below fat/scarpa's fascia<br>• A clinical diagnosis of intra-abdominal collection

	(fever/abdominal pain) with operative/radiological evidence of a collection
**Cardiac**	All complications newly diagnosed within 30 days of discharge (e.g. AF, MI, etc.), even if unrelated to primary admission
**DVT/PE**	Radiologically confirmed within 30 days of discharge
**Radiological drain**	Any additional procedure after operation, including image guided aspiration of collection or placement of a drain.
**Reoperation**	Any return to theatre for a general surgical cause within 30 days of discharge
**Unplanned HDU/ITU admission**	Any unplanned episodes, even if unrelated to primary presentation

Q12b. Classification of complication using Clavien-Dindo Classification. Graded 1-5 – see Appendix C.

Q13. Care level on discharge. Classed as level of social care input after admission. Please indicate <u>only one</u>. If patient at own home with daily care input please indicate the number of times each day carers attend. If patient is discharged to intermediate care then please record the place of discharge from intermediate care.

Q14. 90 day mortality – counted in whole days, rounded up to the nearest day

Q15. ICU/HDU stay – counted in whole days, rounded up to the nearest day

Q16. Intermediate care length of stay - counted in whole days, rounded up to the nearest day
