# Supplementary material for: Influence of frailty in older patients undergoing emergency laparotomy: a UK-based observational study
Source: BMJ Open. 2017 Oct 6;7(10):e017928. doi: 10.1136/bmjopen-2017-017928 (PMC5640048; doi:10.1136/bmjopen-2017-017928)
Supplement: Author's manuscript [file bmjopen-2017-017928.draft_revisions.pdf]

BMJ Open is committed to open peer review. As part of this commitment we make the peer review history of every article we publish publicly available.

When an article is published we post the peer reviewers' comments and the authors' responses online. We also post the versions of the paper that were used during peer review. These are the versions that the peer review comments apply to.

The versions of the paper that follow are the versions that were submitted during the peer review process. They are not the versions of record or the final published versions. They should not be cited or distributed as the published version of this manuscript.

BMJ Open is an open access journal and the full, final, typeset and author-corrected version of record of the manuscript is available on our site with no access controls, subscription charges or pay-per-view fees (http://bmjopen.bmj.com).

If you have any questions on BMJ Open's open peer review process please email editorial.bmjopen@bmj.com

BMJ Open

A NATIONAL MULTI-CENTRE STUDY ASSESSING THE INFLUENCE OF FRAILTY IN OLDER PATIENTS UNDERGOING EMERGENCY LAPAROTOMY

Journal:	*BMJ Open*
Manuscript ID	bmjopen-2017-017928
Article Type:	Protocol
Date Submitted by the Author:	31-May-2017
Complete List of Authors:	Hewitt, Jonathan; Cardiff University, Geriatric Medicine
**Primary Subject Heading**:	Surgery
Secondary Subject Heading:	Geriatric medicine, Epidemiology
Keywords:	Emergency, Older People, Frailty, Laparotomy

SCHOLARONE™ Manuscripts

For peer review only

SURGERY PROTOCOL

A NATIONAL MULTI-CENTRE STUDY ASSESSING THE INFLUENCE OF FRAILTY IN OLDER PATIENTS UNDERGOING EMERGENCY LAPAROTOMY

AUTHORS LIST:

THE ELF STEERING COMMITTEE ON BEHALF OF THE NORTH WEST RESEARCH COLLABORATIVE (NWRC)

THE OLDER PERSONS SURGICAL OUTCOMES COLLABORATIVE (OPSOC)

THE NORTH WEST SURGICAL TRIALS CENTRE

CORRESPONDING AUTHOR, JONATHAN HEWITT, HEWITTJ2@CARDIFF.AC.UK

DECLARATION: *We have read and understood BMJ policy on declaration of interests and declare no conflicts of interest.*

THIS RESEARCH RECEIVED NO SPECIFIC GRANT FROM ANY FUNDING AGENCY IN THE PUBLIC, COMMERCIAL OR NOT-FOR-PROFIT SECTORS

For peer review only

ABSTRACT

Introduction

The National Emergency Laparotomy Audit (NELA) has reported that older patients (65 years and over) form a large percentage of emergency high-risk cases with increased post-operative morbidity and mortality. With the population continuing to age rapidly it is clear a greater understanding of the factors affecting surgical outcomes in older patients is required. Frailty is a relatively new concept taking into account a variety of factors that increases an individual's vulnerability to increased dependency and death. Research has suggested that high frailty scores increase post-operative complications, length of stay and mortality but the majority of these studies have been carried out on elective patients. Knowledge of how frailty affects patients in an emergency setting would aid clinicians' and patients' decision-making process.

Methods and analysis

This multicentre study will include consecutive adult patients aged 65 years and over undergoing emergency laparotomies over a 3-month period at 52 NHS hospitals across the UK. The primary outcome will be 90 day mortality. Secondary outcomes will include; length of hospital stay, 30 day complications, change in level of independence and 30 day readmission. This study has been powered to detect a 10% change in mortality associated with frailty (n=500 patients).

Ethics and dissemination

This study has been approved by the National Health Service Research Ethics Committee. It has been registered centrally with HRA for English sites, NRSPCC for Scottish sites and Health and Care Research Permissions Service for sites in Wales. This study is also registered online at www.clinicaltrials.gov (registration number NCT02952430)

Dissemination will be via international and national surgical and geriatric conferences. In addition, manuscripts will be prepared following the close of the project.

For peer review only

BACKGROUND

The population is ageing. This has implications for health care provision, including surgery(1). The second report of The National Emergency Laparotomy Audit (NELA) in the UK found that over half of patients undergoing major emergency general surgical procedures were older adults (65 years and above) with the highest risk, longest length of stay and highest mortality. NELA had previously recommended input for older adults by elderly medicine specialists from findings in their first report, but this was only reported in 10% of all cases (2). Clinical decision making in older patients can be difficult as they have the unique challenges of multi-morbidity, polypharmacy and cognitive impairment which can occur separately or more commonly in combination. Several risk stratification methods exist to aid the surgical and anaesthetic team, but are limited as they are generally extrapolated from cohorts of much younger patients. A greater understanding of factors involved in surgical outcomes in older patients is therefore required (3).

Frailty is defined as 'a medical syndrome with multiple causes and contributors that is characterised by diminished strength, endurance, and reduced physiologic function that increases an individual's vulnerability for developing increased dependency or death' (4). This definition is now commonplace in Geriatric medicine with frailty routinely assessed in every hospital in the UK with Older Peoples services.

Assessment of frailty in emergency surgery has been assessed in a limited number of studies. Of those, high frailty scores pre-operatively correlate with increased post-operative complications, length of stay, 30 and 90-day mortality and likelihood of institutionalisation (5,6). However, there is substantial methodological heterogeneity with few studies focusing solely on older patients, being prospective in design and including all surgical patients admitted to an acute surgical ward, rather only those undergoing emergency laparotomy. Knowledge of how frailty affects outcomes after emergency laparotomy will aid surgeons in decision making in this complex group of patients but, most importantly, help to inform the consent process for patients and their families.

Aims

For peer review only

To assess whether pre-operative frailty correlates with outcomes in older surgical patients undergoing emergency laparotomy (Emergency Laparotomy and Frailty – The ELF study)

METHODS

Study design

A multicentre observational study.

Study setting

Hospitals in the UK that provide emergency general surgery have been invited to participate. Fifty two hospitals have expressed interest in taking part in the audit. Research will be conducted using the established surgical and geriatric registrar led research networks(7, 8) The methodology for these networks is well described but in brief the networks provide a centrally coordinated research network that promoted and advertised the ELF study. Potential collaborators were invited to take part in data collection, via a standard expression of interest application. The central study team (described below) subsequently provided the ethical approval, protocol, central organisation and long term delivery of the project. Support was provided by the North West Surgical Trials Centre (www.nwstc.org.uk).

ELF Steering Committee

The steering committee comprises surgical trainees, consultant general surgeons, interested in outcomes for older people undergoing surgery. It is formed from two established research groups, the North West Research Collaborative and the Older Persons Surgical Outcomes Collaboration (OPSOC). The steering committee is responsible for protocol design, data handling, analysis, dissemination of results and the preparation of manuscripts. The ELF steering committee is responsible for the use of data resulting from this project.

Principal investigators

The Principal Investigators at each participating site are responsible for organising and leading the local ELF teams. They have submitted relevant documents to local Research and Development departments for approval and ensure that collaborators

For peer review only

act in accordance with local clinical governance and guidelines. These local leads act as a link between the local ELF team and the ELF steering committee. They are the first point of contact for local collaborators and are responsible for the dissemination of information to local collaborators from the ELF steering committee.

Inclusion criteria

- Patients aged 65 years and over
- Patients who undergo an expedited, urgent or emergency abdominal procedure on the gastrointestinal tract, including the following:
- Open, laparoscopic or laparoscopic-assisted procedures
- Procedures involving the stomach, small or large bowel, or rectum for conditions such as perforation, ischaemia, abdominal abscess, bleeding or obstruction
- Washout/evacuation of intra-peritoneal abdominal abscess (unless due to appendicitis or cholecystitis – excluded, see below)
- Washout/evacuation of intra-peritoneal abdominal haematoma
- Bowel resection/repair due to incarcerated umbilical, inguinal and femoral hernias (but not hernia repair without bowel resection/repair)
- Bowel resection/repair due to obstructing/incarcerated incisional hernias provided the presentation and findings were acute
- Laparotomy or laparoscopy with inoperable pathology (i.e. peritoneal/ hepatic metastases)
- Laparoscopic/open adhesiolysis
- Return to theatre for repair of substantial dehiscence of major abdominal wound (i.e. "burst abdomen") or any major post-operative complication (including all operations meeting the above criteria occurring as a complication of previous non-GI surgery, specific examples available at www.nela.org.uk/criteria)

Exclusion criteria

- Frailty score not documented on pre-operative admission clerking
- Elective laparotomy/laparoscopy

For peer review only

- Diagnostic laparoscopy/laparotomy where no further procedure is performed (N.B. if no procedure is performed because of inoperable pathology, then include)
- Appendicectomy +/- drainage of localised collection, unless the procedure is incidental to a non-elective procedure on the GI tract
- Cholecystectomy +/- drainage of localised collection, unless the procedure is incidental to a non-elective procedure on the GI tract
  (All surgery involving the appendix or gallbladder, including any surgery relating to complications such as abscess or bile leak is excluded. The only exception to this is if carried out as an incidental procedure to a more major procedure)
- Non-elective hernia repair without bowel resection
- Minor abdominal wall dehiscence unless causing bowel complication requiring resection
- Vascular surgery
- Caesarean section or obstetric laparotomies
- Gynaecological laparotomy (however bowel resection performed as non-elective procedure for obstruction due to cancer would be included)
- Ruptured ectopic pregnancy, or pelvic abscesses due to pelvic inflammatory disease
- Laparotomy/laparoscopy for pathology caused by blunt or penetrating trauma
- All surgery relating to organ transplantation (including returns to theatre for any reason following transplant surgery)
- Surgery relating to sclerosing peritonitis
- Surgery for removal of dialysis catheters
- Laparotomy/laparoscopy for oesophageal pathology
- Laparotomy/laparoscopy for pathology of the spleen, renal tract, kidneys, liver, gall bladder and biliary tree, pancreas or urinary tract

Patient identification and data collection

Patients will be screened for inclusion criteria by the local team. Data collection will be carried out using the case report form presented in Appendix A. Hospital or NHS number will not be entered into this form but will be kept separately with a key sheet.

For peer review only

Basic demographics, comorbidities and polypharmacy data will be recorded. Co-morbidities will be collected based upon the Charlson Co-morbidity Index, a validated measure of prognostic impact of multiple chronic illnesses (9). This will allow for standardisation of comparisons between any groups. Data will also be collected on baseline independence status, assessed by the number of times social services provide care (1-4 times), and living in a residential or nursing home, measured both pre- and post-discharge.

Frailty will be measured using the Clinical Frailty Score (Appendix B). This has been validated for use to assess frailty in older general surgical patients and OPSOC has successfully applied this before in previous work in this area(10). The score ranks from 1 to 7 with a score of ≥5 being classed as frail.

Data will be collected on pre-operative risk from scoring systems used commonly within emergency general surgery. This will include the P-POSSUM score (11) and the American Society of Anaesthesiologist grade (ASA) (12).

Data will be collected on operative procedures performed. Information will be obtained from patient case notes on 30 day outcomes. This includes 30-day mortality and evidence of post-operative complications. These complications will be rated using the Clavien-Dindo classification (Appendix C). This will allow for complications to be rated and outcomes to be assessed together. Finally information will be obtained from the patient notes regarding 90 day mortality.

Timetable

**Period**	**Date**
Case identification period	20/03/2017 - 19/06/2017
Data collection completion date	19/09/2017
Validation completion date	30/09/2017

For peer review only

Primary outcome

90 day mortality

Secondary outcomes

- Length of hospital stay (measured in days)
- Post-operative complications (yes/no and Clavien-Dindo grade of complication)
- Change in level of independence
- Length of stay on HDU and ICU (measured in days)
- Intermediate care stay on discharge (yes/no and duration of stay measured in length of days)
- 30 day mortality
- 30 day re-admission

Quality assurance

The study has been registered (www.clinicaltrials.gov, registration number NCT02952430)

The quality of this study has been assessed by the following means:

- Steering group meetings: 03/10/2016 and 13/12/2016
- Review by OPSOC
- Peer review by professionals with relevant expertise (Clinical trialists, statisticians, surgeons and geriatricians)
- Review by Research & Development department at NHS Greater Glasgow & Clyde (Sponsor Institution)
- Review by North West Surgical Trials Centre Trial Adoption Committee

Validation

Data validation will be performed by local teams on 25% of data fields for 10% of cases. The validated fields will include key demographic and outcome data.

Data management

For peer review only

Completed datasets will be entered into an established and specifically designed online secure electronic database [REDCap, www.project-**redcap**.org]. Password-protected login details will be provided to local collaborators permitting secure data entry into the database. All data will be handled in accordance with the Data Protection Act 1998. All transmission and storage of data will be encrypted and compliant with HIPAA security guidelines.

No patient identifiable information will be uploaded or stored on the secure database. Collaborators will anonymise patients by recording patient hospital numbers alongside database numbers in a separate spreadsheet in order to aid the collection of data locally.

Statistical analysis & power calculation

Using OPSOC data, frailty exists in 28% of older patients admitted with emergency surgical conditions. Fifty four percent of the frail people who underwent surgery, had died after 90 days. In order to detect a 10% difference in mortality rate at Day 90 between frail and non frail patients a sample size of 480 is required, given an expected mortality proportion in those not frail of 0.075 and those frail of 0.175 (data from OPSOC), assuming an 80% power. We anticipate minimal patients that are lost to follow-up and to account for this, we will aim to recruit 500 patients.

Statistical support will be provided by OPSOC. Data will be analysed for correlation between frailty and post-operative outcomes, including 90 day mortality, complications and loss of independence.

Our primary analysis will be a logistic regression of 90 day mortality by frailty, adjusted for age (65 to 74, and over 75 years old) and gender. We will carry out a secondary analysis of the primary outcome, by including additional clinical mediators which are determined statistically important using a likelihood ratio test with a step-wise model fitting approach of nested regression models, and presented as a final multivariable model. All analyses will be presented as adjusted Odds Ratio with associated 95% confidence intervals and p-values.

All other outcomes will be analysed as per the above analysis, but will be deemed secondary outcomes.

For peer review only

Anticipated recruitment

Data will be collected at participating sites for all patients meeting the inclusion criteria over a three-month period. This has been calculated based on information submitted by participating sites regarding the number of laparotomies performed per month on patients aged 65 and over. According to this data, three months should permit the identification of 500 patients.

ETHICS AND DISSEMINATION

Ethical approval

Ethical approval for this study was granted by a National Health Service Research Ethics Committee via the Proportionate Review Service. This was granted by the Black Country Research Committee on 28th November 2016 (REC Reference 16/WM/0500). The same committee reviewed the amended protocol and granted a favourable opinion on 6th February 2017.

Registration

This study has been registered, reviewed and approved by the following organisations:

- The HRA (Health Research Authority) for sites in England
- The NRSPCC (NHS Research Scotland Permissions Co-ordinating Centre) for sites in Scotland
- The Health & Care Research Permissions Service for sites in Wales

All participating units must obtain approval from their local Research & Development department consistent with the guidance from their relevant national organisation:

- The HRA (Health Research Authority) for sites in England
- The NRSPCC (NHS Research Scotland Permissions Co-ordinating Centre) for sites in Scotland
- The Health & Care Research Permissions Service for sites in Wales

For peer review only

The project will therefore be registered locally with the Trust Research & Development department prior to commencing patient identification and data collection at each site. It is the responsibility of the local ELF team to ensure that local Research & Development approvals are in place prior to commencing data collection.

Dissemination

All data will be reported as a whole cohort. Unit level data for comparison will be fed back to collaborators to support local service improvement. This project will be submitted for presentation at a national or international surgical and geriatric conference.

Manuscript(s) will be prepared following close of the project.

For peer review only

REFERENCES

1. Partridge JS, *et al.*. Frailty in the older surgical patient: A review. *Age and ageing* 2012;41(2):142-7

2. NELA project team. Second Patient report of the National Emergency Laparotomy Audit. *RCoA London* 2016 http://www.nela.org.uk/reports

3. Lees MC, *et al*.. Perioperative factors predicting poor outcome in elderly patients following emergency general surgery: a multivariate regression analysis. *Can J Surg.* 2015;58(5):312-7

4. Morley JE, *et al.* Frailty Consensus: A call to action. *J Am Med Dir Assoc*. 2013;14:392-7

5. Hewitt J, *et al.* Prevalence of frailty and its association with mortality in general surgery. *Am J Surg.* 2015;209:254-9

6. Farhat JS, *et al.* Are the frail destined to fail? Frailty index as a predictor of surgical morbidity and mortality in the elderly. *J Trauma Acute Care Surg.* 2012;72(6):1526-31

7. Dowsell G, *et al.* How to set up and manage a trainee-led research collaborative. *BMC Med Educ*. 2014 May 14:14:94 doi:10. 1186/1472-6920-14-94

8. Jelley B, *et al.*. Cohort profile: the Welsh Geriatric Registrar-Led Research Network (WeGeN): rationale, design and description. *BMJ Open.* 2017 Feb 14;7(2):e013031. doi: 10.1136/bmjopen-2016-013031

9. Charlson M, *et al*.. A new method of classifying prognostic comorbidity in longitudinal studies: Development and validation. Journal of Chronic Diseases. 1987 40:5:373-378

For peer review only

10. Rockwood K, *et al.* A global clinical measure of fitness and frailty in elderly people. *CMAJ.* 2005;173:489-95

11. Copeland GP, *et al.*. POSSUM: A scoring system for surgical audit. *Br J Surg*. 1991;78:355–60.

12. Dripps RD. New classification of physical status. *Anesthesiol*. 1963;24:111.

For peer review only

Authors Contributions. This is a group collaborative authorship manuscript

THE ELF STEERING COMMITTEE ON BEHALF OF THE NORTH WEST RESEARCH COLLABORATIVE (NWRC).

This team of 6 surgical trainees contributed to study conception, design and development, including the ethical application.

THE OLDER PERSONS SURGICAL OUTCOMES COLLABORATIVE (OPSOC)

This team have extensive knowledge of surgical disease in the Older Person. The project built and direct led on from their previous work. This team mentored the project and have commented on all aspects of the study design and materials and will form an integral part of the writing group. Jonathan Hewitt is the national OPSOC lead and led the development of the protocol manuscript for submission. He is named as corresponding author to facilitate institutional payment (via his employer, Cardiff University) for the open access fee. He takes no greater authorship credit than all the fellow group authors.

THE NORTH WEST SURGICAL TRIALS CENTRE

This is the local research support centre for the North West Research Collaborative. They have provided support and guidance about all technical aspects of study set up. They have also provided the computer and database support to the study.

CORRESPONDING AUTHOR, JONATHAN HEWITT, HEWITTJ2@CARDIFF.AC.UK

Funding

This study received no external funding

Conflict of Interests

There are no conflict of interests to declare.

For peer review only

Appendix A: Case Report Form

Q1	Study ID	
Q2	Age at admission to study (years)	
Q3	Sex	Male Female
Q4	Comorbidities	CCF Y/N COPD Y/N<br>CVA Y/N Dementia Y/N<br>Hemiplegia Y/N CKD Y/N<br>Leukaemia Y/N DM(complicated) Y/N<br>Lymphoma Y/N<br>DM(uncomplicated) Y/N Mild liver disease Y/N IHD Y/N<br>Severe liver disease Y/N PVD Y/N<br>Solid tumour Y/N Metastatic tumour Y/N<br>AIDS Y/N Peptic ulcer disease Y/N<br>Connective tissue disease Y/N<br>Other:____________________
Q5	Polypharmacy (≥5 medications)	Yes No
Q6	Care level prior to admission*<br><br>**The term "carer" to include both formal and informal care arrangements i.e. friends/ relatives*	Home (No carers*)<br>Home (with carers* _____ times/day)<br>Residential Home<br>Nursing home<br>Intermediate care<br>Other: ____________________
Q7	Frailty score	
Q8	Interval between admission & surgery (days)	

For peer review only

Q9a	Pre-operative ASA grade	
Q9b	Pre-operative PPOSSUM	Morbidity: Mortality:
Q10a	Operative indication	Peritonitis Perforation<br>Abdominal abscess Anastomotic leak<br>Intestinal fistula Sepsis (other)<br>Intestinal obstruction Haemorrhage<br>Ischaemia Colitis<br>Abdominal wound dehiscence<br>Abdominal compartment syndrome<br>Planned relook<br>Other ___________________________
Q10b	Procedure (circle **ALL** that apply)	Peptic ulcer (suture or repair of perforation)<br>Peptic ulcer (oversew of bleeding)<br>Gastric surgery - other<br>Small bowel resection<br>Colectomy: Left (including anterior resection)<br>Right Subtotal<br>Hartmann's procedure<br>Colorectal resection – other<br>Haemostasis Enterotomy<br>Stoma formation Stoma revision<br>Adhesiolysis Intestinal bypass<br>Reduction of volvulus Washout only

For peer review only

		Abdominal wall closure<br>Drainage of abscess/collection<br>Laparostomy formation<br>Repair of intestinal perforation<br>Resection of other intra-abdominal tumours<br>Exploratory/ re-look laparotomy only<br>Not amenable to surgery<br>Other ___________________________
Q10c	Primary procedure type	Open<br>Laparoscopic<br>Laparoscopic converted to open<br>Laparoscopic-assisted
Q11a	Length of stay (days)	
Q11b	Readmission to hospital within 30 days	Yes No
Q11c	Reason for readmission	
Q12a	Post-operative complication within 30 days	Yes No
Q12b	Grade of complication	
Q13	Care level on discharge<br>**The term "carer" to include both formal and informal care arrangements i.e. friends/ relatives*	Home (No carers*)<br>Home (with carers* _____ times/day)<br>Residential Home<br>Nursing home<br>Other: ______________________________
Q14	90 day mortality	Yes No
Q15	Length of ICU/HDU stay	ICU HDU<br>Days total stay ___________
Q16	Intermediate care stay (days)	Yes No

For peer review only

		Days total stay ___________

For peer review only

Appendix B: Canadian Study of Health and Ageing (CSHA) Frailty Score (Rockwood Score)

The CSHA Frailty Scale	
**1 – Very fit**	Robust, active, energetic, well-motivated and fit; these people commonly exercise regularly and are in the most fit group for their age.
**2 – Well**	Without active disease, but less fit than people in category 1.
**3 – Well, with treated comorbid disease**	Disease symptoms are well controlled compared with those in category 4.
**4 – Apparently vulnerable**	Although not frankly dependent, these people commonly complain of being "slowed up" or have disease symptoms.
**5 – Mildly frail**	With limited dependence on others for instrumental* activities of daily living.
**6 – Moderately frail**	Help is needed with both instrumental* and non-instrumental activities of daily living.
**7 – Severely frail**	Completely dependent on others for activities of daily living, or terminally ill.

- *Non-instrumental activities of daily living are basic everyday tasks such as walking, bathing, dressing, toileting, brushing teeth and eating. Instrumental activities of daily living are further tasks such as cooking, shopping, driving etc. Further explanation is available at the following link if required:*

  https://asourparentsage.net/2009/12/17/adls-and-iadls-whats-the-difference/

For peer review only

For peer review only

Appendix C:

Clavien-Dindo Classification of Surgical Complications

Clavien-Dindo Classification of Surgical Complications	
**Grade I**	Any deviation from the normal postoperative course without the need for pharmacological treatment or surgical, endoscopic, and radiological interventions. Allowed therapeutic regimens are: drugs such as antiemetics, antipyretics, analgetics, diuretics, electrolytes, and physiotherapy. This grade also includes wound infections opened at the bedside.
**Grade II**	Requiring pharmacological treatment with drugs other than allowed for grade I complications. Blood transfusions and total parenteral nutrition are also included.
**Grade IIIa**	Surgical, endoscopic, or radiological intervention that is not under general anesthesia
**Grade IIIb**	Surgical, endoscopic, or radiological intervention that is under general anesthesia
**Grade IVa**	Life-threatening complication requiring intermediate care or intensive care unit management, single organ dysfunction (including dialysis, brain hemorrhage, ischemic stroke, and subarrachnoidal bleeding)
**Grade IVb**	Life-threatening complication requiring intermediate care or intensive care unit management, multi-organ dysfunction (including dialysis)
**Grade V**	Death of a patient
**Suffix "d"**	If the patient suffers from a complication at the time of discharge, the suffix "d" (for "disability") is added to the respective grade of complication. This label indicates the need for a follow-up to fully evaluate the complication

w only

Appendix D: Definitions

Day of study – this is defined by the number of 24h periods passed from first attendance at hospital, the event being examined (e.g. date of operation). Patients admitted and the event occurs with the first 24hrs are classed as 1 day.

Q1. Combination of centre Number and record number. For example if you are in centre 024 and recording data on the 35th patient, the number would be 024.35

Q2. Age in completed years on date of admission to hospital

Q3. Please indicate sex of patient

Q4. These are comorbidities as defined by the Charlson Comorbidity Index. Each should be marked as present if there is any previous documented history of each diagnosis.

**Myocardial infarct**	History of medically documented myocardial infarction
**Congestive heart failure**	Symptomatic congestive heart failure w/ response to specific treatment
**Peripheral vascular disease**	Intermittent claudication, peripheral arterial bypass for insufficiency, gangrene, acute arterial insufficiency, untreated aneurysm (≥6cm)
**Cerebrovascular disease (except hemiplegia)**	History of TIA, or CVA with no or minor sequelae
**Dementia**	Chronic cognitive deficit
**Chronic pulmonary disease**	Symptomatic dyspnoea due to chronic respiratory conditions (inc. asthma)
**Connective tissue disease**	SLE, polymyositis, polymyalgia rheumatic, moderate to severe rheumatoid arthritis
**Peptic ulcer disease**	Patients who have required treatment for peptic ulcer disease
**Mild liver disease**	Cirrhosis without portal hypertension, chronic hepatitis
**Diabetes**	Diabetes with medication

For peer review only

**(without complication)**	
**Diabetes with end organ damage**	Retinopathy, neuropathy, nephropathy
**Hemiplegia (or paraplegia)**	Hemiplegia or paraplegia
**Moderate of severe renal disease**	Creatinine>265umol/L, dialysis, transplantation, uraemic syndrome
**Solid tumour (non-metastatic)**	Initially treated in the last 5 years exclude non-melanomatous skin cancers and in situ cervical carcinoma
**Leukaemia**	CML, CLL, AML, ALL, PV
**Lymphoma, Multiple myeloma**	Non-Hodgkin's Lymphoma, Hodgkin's, Waldenström, multiple myeloma
**Moderate or severe liver disease**	Cirrhosis with portal hypertension +/- variceal bleeding
**Metastatic solid tumour**	Metastatic solid tumour
**AIDS**	AIDS & AIDS-related complex

Q5. Polypharmacy counted as five or more prescribed regular medications on admission. This includes regular eye drops, inhalers and analgesia.

Q6. Care level prior to admission. Classed as level of social care input prior to admission. Please indicate only one. If patient at own home with daily care input please indicate the number of times each day carers attend.

Q7. Frailty score, 1-7 using the modified Rockwood Scale (please see Appendix B)

Q8. Interval between admission and emergency procedure. Classed as whole days, rounded up to the nearest whole day. (e.g. 0-24h classed as 1 day, 24-48h classed as 2 days)

Q9a. American Society of Anaesthesiologist (ASA) grade:

For peer review only

Grade	Description
**1**	Healthy individual with no systemic disease
**2**	Mild systemic disease not limiting activity
**3**	Severe systemic disease that limits activity but is not incapacitating
**4**	Incapacitating systemic disease which is constantly life-threatening
**5**	Moribund, not expected to survive 24 hours with or without surgery

Q9b. P-POSSUM Score: Calculated from pre-morbid status using multiple markers of baseline function including age, cardiac status, observations and blood test results. This should already be routinely documented in all patient notes as part of the National Emergency Laparotomy Audit dataset. If required please use the calculator found at http://www.riskprediction.org.uk/index-pp.php

Q10a. Primary operative indication as per National Emergency Laparotomy Audit data collection

Q10b. Primary operative procedure as per National Emergency Laparotomy Audit collection tool

Q10c. Primary surgical method used during the procedure. NOTE: Laparoscopic assisted should be used if decision to proceed to open was part of the pre-operative procedure planning, otherwise laparoscopic converted to open should be used.

Q11a. Total length of stay of primary admission is defined as number of 24h periods or part thereof, passed from first attendance at hospital, to discharge. Patients admitted and discharged with the first 24hrs are not included in the study, between 24-48hrs 2 day etc.

Q11b. Readmission to hospital within 30 days as an emergency (classed as whole days, rounded up to the nearest whole day) regardless of cause.

For peer review only

Q11c. Reason for readmission to hospital

Q12a. Post-operative complications include:

**Abdominal wall dehiscence**	Full thickness dehiscence of laparotomy wound within 30 days of discharge
**Anastomotic leakage**	A clinical diagnosis will require symptoms related to leakage (gas, pus, or faecal discharge from the drain site, peritonitis or discharge of pus from the rectum). In the event of a clinically suspicious leak (fever or abdominal pain) the diagnosis can be established by operative or radiological diagnosis. When an anastomosis is defunctioned the presence or absence of a leak will be established by contrast radiology.
**Urinary tract infection**	Patient needs to meet two of the following criteria:<br>• Fever >38^{0}C<br>• Suprapubic tenderness<br>• Costovertebral angle pain or tenderness<br>• Urinary urgency<br>• Urinary frequency<br>• Dysuria<br>• Urine culture with no more than two species of organisms identified, at least one of which is a bacterium of ≥105 CFU/mL
**Pneumonia**	Patient must meet one of the following criteria:<br>• Dullness to percussion on physical examination of chest and any of the following:<br>- New onset of purulent sputum or change in character of sputum<br>- Organism isolated from blood culture<br>- Isolation of pathogen from specimen obtained from transtracheal aspirate,

For peer review only

	bronchial brushing or biopsy<br>• Chest radiographic examination shows new or progressive infiltrate, consolidation, cavitation or pleural effusion and any of the following<br>- New onset of purulent sputum or change in character of sputum<br>- Organism isolated from blood culture<br>- Isolation of pathogen from specimen obtained from transtracheal aspirate, bronchial brushing or biopsy<br>- Isolation of virus or detection of viral antigen in respiratory secretions<br>- Diagnostic single antibody titre (IgM) or four-fold increase in paired serum samples (IgG) for pathogen
**Superficial SSI**	Patient must meet one of these criteria<br>• Purulent drainage from the incision<br>• At least two of – pain, localised swelling, redness, head, fever AND the incision is opened deliberately to manage infection or the clinician diagnoses a SSI<br>• Wound organisms AND pus cells from aspirate/swab
**Deep (intra-abdominal) SSI**	Patient must meet one of these criteria<br>• A clinical diagnosis of wound infection with dehiscence of mass closure or any layer below fat/scarpa's fascia<br>• A clinical diagnosis of intra-abdominal collection (fever/abdominal pain) with operative/radiological evidence of a collection
**Cardiac**	All complications newly diagnosed within 30 days of discharge (e.g. AF, MI, etc.), even if unrelated to primary admission

For peer review only

**DVT/PE**	Radiologically confirmed within 30 days of discharge
**Radiological drain**	Any additional procedure after operation, including image guided aspiration of collection or placement of a drain.
**Reoperation**	Any return to theatre for a general surgical cause within 30 days of discharge
**Unplanned HDU/ITU admission**	Any unplanned episodes, even if unrelated to primary presentation

Q12b. Classification of complication using Clavien-Dindo Classification. Graded 1-5 – see Appendix C.

Q13. Care level on discharge. Classed as level of social care input after admission. Please indicate only one. If patient at own home with daily care input please indicate the number of times each day carers attend. If patient is discharged to intermediate care then please record the place of discharge from intermediate care.

Q14. 90 day mortality – counted in whole days, rounded up to the nearest day

Q15. ICU/HDU stay – counted in whole days, rounded up to the nearest day

Q16. Intermediate care length of stay - counted in whole days, rounded up to the nearest day

For peer review only

THE INFLUENCE OF FRAILTY IN OLDER PATIENTS UNDERGOING EMERGENCY LAPAROTOMY, A UK BASED OBSERVATIONAL STUDY

Journal:	*BMJ Open*
Manuscript ID	bmjopen-2017-017928.R1
Article Type:	Protocol
Date Submitted by the Author:	24-Jul-2017
Complete List of Authors:	Parmar, Kat; Wythenshawe Hospital<br>Pearce, Lyndsay; Manchester Royal Infirmary, Department of General Surgery<br>Farrell, Ian; Manchester Royal Infirmary, Department of General Surgery<br>Hewitt, Jonathan; Cardiff University, Geriatric Medicine<br>Moug, Susan; Royal Alexandra Hospital, General Surgery
**Primary Subject Heading**:	Surgery
Secondary Subject Heading:	Geriatric medicine, Epidemiology
Keywords:	Emergency, Older People, Frailty, Laparotomy

SCHOLARONE™ Manuscripts

For peer review only

For peer review only - http://bmjopen.bmj.com/site/about/guidelines.xhtml

SURGERY PROTOCOL

THE INFLUENCE OF FRAILTY IN OLDER PATIENTS UNDERGOING EMERGENCY LAPAROTOMY, A UK BASED OBSERVATIONAL STUDY

AUTHORS LIST: PARMAR KL, PEARCE L, FARRELL I, HEWITT J, MOUG SJ.

THE ELF STEERING COMMITTEE ON BEHALF OF THE NORTH WEST RESEARCH COLLABORATIVE (NWRC)

THE OLDER PERSONS SURGICAL OUTCOMES COLLABORATIVE (OPSOC)

THE NORTH WEST SURGICAL TRIALS CENTRE

CORRESPONDING AUTHOR, JONATHAN HEWITT, HEWITTJ2@CARDIFF.AC.UK

DECLARATION: *We have read and understood BMJ policy on declaration of interests and declare no conflicts of interest.*

THIS RESEARCH RECEIVED NO SPECIFIC GRANT FROM ANY FUNDING AGENCY IN THE PUBLIC, COMMERCIAL OR NOT-FOR-PROFIT SECTORS

CONTRIBUTION STATEMENT: THIS IS A COLLABORATIVE PUBLICATION. KLP, LP, JH AND SJM, CONCEIVED AND DEVELOPED THE PROJECT. IF AND JH WROTE THE FIRST DRAFT OF THE MANUSCRIPT. ALL AUTHORS CONTRIBUTED TO SUBSEQUENT DRAFTS OF THE MANUSCRIPT.

For peer review only

ABSTRACT

Introduction

The National Emergency Laparotomy Audit (NELA) has reported that older patients (65 years and over) form a large percentage of emergency high-risk cases with increased post-operative morbidity and mortality. With the population continuing to age rapidly it is clear a greater understanding of the factors affecting surgical outcomes in older patients is required. Frailty is a relatively new concept taking into account a variety of factors that increases an individual's vulnerability to increased dependency and death. Research has suggested that high frailty scores increase post-operative complications, length of stay and mortality but the majority of these studies have been carried out on elective patients. Knowledge of how frailty affects patients in an emergency setting would aid clinicians' and patients' decision-making process.

Methods and analysis

This multicentre study will include consecutive adult patients aged 65 years and over undergoing emergency laparotomies over a 3-month period at 52 NHS hospitals across the UK. The primary outcome will be 90 day mortality. Secondary outcomes will include; length of hospital stay, 30 day complications, change in level of independence and 30 day readmission. This study has been powered to detect a 10% change in mortality associated with frailty (n=500 patients).

Ethics and dissemination

This study has been approved by the National Health Service Research Ethics Committee. It has been registered centrally with HRA for English sites, NRSPCC for Scottish sites and Health and Care Research Permissions Service for sites in Wales. This study is also registered online at www.clinicaltrials.gov (registration number NCT02952430)

Dissemination will be via international and national surgical and geriatric conferences. In addition, manuscripts will be prepared following the close of the project.

For peer review only

STRENGTHS AND LIMITATIONS

A large scale multisite study based in the UK

Data collated using the established and effective registrar led research networks

Frailty collated using the Clinical Frailty Scale, which is quick and simple to use

The Clinical Frailty Scale was the only frailty measure collected, a potential limitation

For peer review only

BACKGROUND

The population is ageing. This has implications for health care provision, including surgery(1, 2). The second report of The National Emergency Laparotomy Audit (NELA) in the UK found that over half of patients undergoing major emergency general surgical procedures were older adults (65 years and above) with the highest risk, longest length of stay and highest mortality. NELA had previously recommended input for older adults by elderly medicine specialists from findings in their first report, but this was only reported in 10% of all cases (3). Clinical decision making in older patients can be difficult as they have the unique challenges of multi-morbidity, polypharmacy and cognitive impairment which can occur separately or more commonly in combination. Several risk stratification methods exist to aid the surgical and anaesthetic team, but are limited as they are generally extrapolated from cohorts of much younger patients. A greater understanding of factors involved in surgical outcomes in older patients is therefore required(4).

Frailty is defined as ‘a medical syndrome with multiple causes and contributors that is characterised by diminished strength, endurance, and reduced physiologic function that increases an individual’s vulnerability for developing increased dependency or death’ (5). This definition is now commonplace in Geriatric medicine with frailty routinely assessed in every hospital in the UK with Older Peoples services.

Assessment of frailty in emergency surgery has been assessed in a limited number of studies. Of those, high frailty scores pre-operatively correlate with increased post-operative complications, length of stay, 30 and 90-day mortality and likelihood of institutionalisation (6-8). However, there is substantial methodological heterogeneity with few studies focusing solely on older patients, being prospective in design and including all surgical patients admitted to an acute surgical ward, rather only those undergoing emergency laparotomy. Knowledge of how frailty affects outcomes after emergency laparotomy will aid surgeons in decision making in this complex group of patients but, most importantly, help to inform the consent process for patients and their families.

Aims

For peer review only

To assess whether pre-operative frailty correlates with outcomes in older surgical patients undergoing emergency laparotomy (Emergency Laparotomy and Frailty – The ELF study)

METHODS

Study design

A multicentre observational study.

Study setting

Hospitals in the UK that provide emergency general surgery have been invited to participate. Fifty two hospitals have expressed interest in taking part in the audit. Research will be conducted using the established surgical and geriatric registrar led research networks(9, 10). The methodology for these networks is well described but in brief the networks provide a centrally coordinated research network that promoted and advertised the ELF study. Potential collaborators were invited to take part in data collection, via a standard expression of interest application. The central study team (described below) subsequently provided the ethical approval, protocol, central organisation and long term delivery of the project. Support was provided by the North West Surgical Trials Centre (www.nwstc.org.uk).

ELF Steering Committee

The steering committee comprises surgical trainees and consultant general surgeons, interested in outcomes for older people undergoing surgery. It is formed from two established research groups, the North West Research Collaborative (surgical trainees) and the Older Persons Surgical Outcomes Collaboration (OPSOC; surgeons and geriatricians). The steering committee is responsible for protocol design, data handling, analysis, dissemination of results and the preparation of manuscripts. The ELF steering committee is responsible for the use of data resulting from this project.

Principal investigators

The Principal Investigators at each participating site are responsible for organising and leading the local ELF teams. They have submitted relevant documents to local

For peer review only - http://bmjopen.bmj.com/site/about/guidelines.xhtml

Research and Development departments for approval and ensure that collaborators act in accordance with local clinical governance and guidelines. These local leads act as a link between the local ELF team and the ELF steering committee. They are the first point of contact for local collaborators and are responsible for the dissemination of information to local collaborators from the ELF steering committee.

Inclusion criteria

- Patients aged 65 years and over
- Patients who undergo an expedited, urgent or emergency abdominal procedure on the gastrointestinal tract, including the following:
- Open, laparoscopic or laparoscopic-assisted procedures
- Procedures involving the stomach, small or large bowel, or rectum for conditions such as perforation, ischaemia, abdominal abscess, bleeding or obstruction
- Washout/evacuation of intra-peritoneal abdominal abscess (unless due to appendicitis or cholecystitis – excluded, see below)
- Washout/evacuation of intra-peritoneal abdominal haematoma
- Bowel resection/repair due to incarcerated umbilical, inguinal and femoral hernias (but not hernia repair without bowel resection/repair)
- Bowel resection/repair due to obstructing/incarcerated incisional hernias provided the presentation and findings were acute
- Laparotomy or laparoscopy with inoperable pathology (i.e. peritoneal/ hepatic metastases)
- Laparoscopic/open adhesiolysis
- Return to theatre for repair of substantial dehiscence of major abdominal wound (i.e. "burst abdomen") or any major post-operative complication (including all operations meeting the above criteria occurring as a complication of previous non-GI surgery, specific examples available at www.nela.org.uk/criteria)

Exclusion criteria

- Frailty score not documented on pre-operative admission clerking
- Elective laparotomy/laparoscopy

For peer review only

- Diagnostic laparoscopy/laparotomy where no further procedure is performed (N.B. if no procedure is performed because of inoperable pathology, then include)
- Appendicectomy +/- drainage of localised collection, unless the procedure is incidental to a non-elective procedure on the GI tract
- Cholecystectomy +/- drainage of localised collection, unless the procedure is incidental to a non-elective procedure on the GI tract
  (All surgery involving the appendix or gallbladder, including any surgery relating to complications such as abscess or bile leak is excluded. The only exception to this is if carried out as an incidental procedure to a more major procedure)
- Non-elective hernia repair without bowel resection
- Minor abdominal wall dehiscence unless causing bowel complication requiring resection
- Vascular surgery
- Caesarean section or obstetric laparotomies
- Gynaecological laparotomy (however bowel resection performed as non-elective procedure for obstruction due to cancer would be included)
- Ruptured ectopic pregnancy, or pelvic abscesses due to pelvic inflammatory disease
- Laparotomy/laparoscopy for pathology caused by blunt or penetrating trauma
- All surgery relating to organ transplantation (including returns to theatre for any reason following transplant surgery)
- Surgery relating to sclerosing peritonitis
- Surgery for removal of dialysis catheters
- Laparotomy/laparoscopy for oesophageal pathology
- Laparotomy/laparoscopy for pathology of the spleen, renal tract, kidneys, liver, gall bladder and biliary tree, pancreas or urinary tract

Patient identification and data collection

Patients will be screened for inclusion criteria by the local team. Data collection will be carried out using the case report form presented in Appendix A. Hospital or NHS number will not be entered into this form but will be kept separately with a key sheet.

For peer review only

Basic demographics, comorbidities and polypharmacy data will be recorded. Co-morbidities will be collected based upon the Charlson Co-morbidity Index, a validated measure of prognostic impact of multiple chronic illnesses **(11).** This will allow for standardisation of comparisons between any groups. Data will also be collected on baseline independence status, assessed by the number of times social services provide care (1-4 times), and living in a residential or nursing home, measured both pre- and post-discharge.

Frailty will be measured using the Clinical Frailty Score (Appendix B). This has been validated for use to assess frailty in older general surgical patients and OPSOC has successfully applied this before in previous work in this area (12). The score ranks from 1 to 7 with a score of ≥5 being classed as frail.

Data will be collected on pre-operative risk from scoring systems used commonly within emergency general surgery. This will include the P-POSSUM score (13) and the American Society of Anaesthesiologist grade (ASA) (14).

Data will be collected on operative procedures performed. Information will be obtained from patient case notes on 30 day outcomes. This includes 30-day mortality and evidence of post-operative complications. These complications will be rated using the Clavien-Dindo classification (Appendix C). This will allow for complications to be rated and outcomes to be assessed together. Finally information will be obtained from the patient notes regarding 90 day mortality.

The timetable for data collection is given in Table 1

**Period**	**Date**
Case identification period	20/03/2017 - 19/06/2017
Data collection completion date	19/09/2017
Validation completion date	30/09/2017

Table 1. Timetable for data collection

For peer review only

Primary outcome

90 day mortality

Secondary outcomes

- Length of hospital stay (measured in days)
- Post-operative complications (yes/no and Clavien-Dindo grade of complication)
- Change in level of independence
- Length of stay on HDU and ICU (measured in days)
- Intermediate care stay on discharge (yes/no and duration of stay measured in length of days)
- 30 day mortality
- 30 day re-admission

Quality assurance

The study has been registered (www.clinicaltrials.gov, registration number NCT02952430)

The quality of this study has been assessed by the following means:

- Steering group meetings: 03/10/2016 and 13/12/2016
- Review by OPSOC
- Peer review by professionals with relevant expertise (Clinical trialists, statisticians, surgeons and geriatricians)
- Review by Research & Development department at NHS Greater Glasgow & Clyde (Sponsor Institution)
- Review by North West Surgical Trials Centre Trial Adoption Committee

Validation

Data validation will be performed by local teams on 25% of data fields for 10% of cases. The validated fields will include key demographic and outcome data.

For peer review only

Data management

Completed datasets will be entered into an established and specifically designed online secure electronic database [REDCap, www.project-**redcap**.org]. Password-protected login details will be provided to local collaborators permitting secure data entry into the database. All data will be handled in accordance with the Data Protection Act 1998. All transmission and storage of data will be encrypted and compliant with HIPAA security guidelines.

No patient identifiable information will be uploaded or stored on the secure database. Collaborators will anonymise patients by recording patient hospital numbers alongside database numbers in a separate spreadsheet in order to aid the collection of data locally.

Statistical analysis & power calculation

Using OPSOC data, frailty exists in 28% of older patients admitted with emergency surgical conditions. Fifty four percent of the frail people who underwent surgery, had died after 90 days. In order to detect a 10% difference in mortality rate at Day 90 between frail and non frail patients a sample size of 480 is required, given an expected mortality proportion in those not frail of 0.075 and those frail of 0.175 (data from OPSOC), assuming an 80% power. We anticipate minimal patients that are lost to follow-up and to account for this, we will aim to recruit 500 patients.

Statistical support will be provided by OPSOC. Data will be analysed for correlation between frailty and post-operative outcomes, including 90 day mortality, complications and loss of independence.

Our primary analysis will be a logistic regression of 90 day mortality by frailty, adjusted for age (65 to 74, and over 75 years old) and gender. We will carry out a secondary analysis of the primary outcome, by including additional clinical mediators which are determined statistically important using a likelihood ratio test with a step-wise model fitting approach of nested regression models, and presented as a final multivariable model. All analyses will be presented as adjusted Odds Ratio with associated 95% confidence intervals and p-values.

For peer review only

All other outcomes will be analysed as per the above analysis, but will be deemed secondary outcomes.

Anticipated recruitment

Data will be collected at participating sites for all patients meeting the inclusion criteria over a three-month period. This has been calculated based on information submitted by participating sites regarding the number of laparotomies performed per month on patients aged 65 and over. According to this data, three months should permit the identification of 500 patients.

ETHICS AND DISSEMINATION

Ethical approval

Ethical approval for this study was granted by a National Health Service Research Ethics Committee via the Proportionate Review Service. This was granted by the Black Country Research Committee on 28th November 2016 (REC Reference 16/WM/0500). The same committee reviewed the amended protocol and granted a favourable opinion on 6th February 2017.

Registration

This study has been registered, reviewed and approved by the following organisations:

- The HRA (Health Research Authority) for sites in England
- The NRSPCC (NHS Research Scotland Permissions Co-ordinating Centre) for sites in Scotland
- The Health & Care Research Permissions Service for sites in Wales

All participating units must obtain approval from their local Research & Development department consistent with the guidance from their relevant national organisation:

- The HRA (Health Research Authority) for sites in England

For peer review only

- The NRSPCC (NHS Research Scotland Permissions Co-ordinating Centre) for sites in Scotland
- The Health & Care Research Permissions Service for sites in Wales

The project will therefore be registered locally with the Trust Research & Development department prior to commencing patient identification and data collection at each site. It is the responsibility of the local ELF team to ensure that local Research & Development approvals are in place prior to commencing data collection.

Dissemination

All data will be reported as a whole cohort. Unit level data for comparison will be fed back to collaborators to support local service improvement. This project will be submitted for presentation at a national or international surgical and geriatric conference.

Manuscript(s) will be prepared following close of the project.

For peer review only

REFERENCES

1. Partridge JS, Harari D, Dhesi JK. Frailty in the older surgical patient: a review. Age and ageing. 2012;41(2):142-7.
2. Soreide K, Wijnhoven BP. Surgery for an ageing population. Br J Surg. 2016;103(2):e7-9.
3. London R. NELA project team. Second Patient report of the National Emergency Laparotomy Audit. 2016 [cited 2017 June]. Available from: http://www.nela.org.uk/reports.
4. Lees MC, Merani S, Tauh K, Khadaroo RG. Perioperative factors predicting poor outcome in elderly patients following emergency general surgery: a multivariate regression analysis. Can J Surg. 2015;58(5):312-7.
5. Morley JE, Vellas B, van Kan GA, Anker SD, Bauer JM, Bernabei R, et al. Frailty consensus: a call to action. J Am Med Dir Assoc. 2013;14(6):392-7.
6. Farhat JS, Velanovich V, Falvo AJ, Horst HM, Swartz A, Patton JH, Jr., et al. Are the frail destined to fail? Frailty index as predictor of surgical morbidity and mortality in the elderly. The journal of trauma and acute care surgery. 2012;72(6):1526-30; discussion 30-1.
7. Hewitt J, Moug S, Middleton M, Chakrabarti M, Stechman M, McCarthy K. Prevalence of frailty and its association with mortality in general surgery. Am J Surg. 2015;209(2):254-9.
8. Orouji Jokar T, Ibraheem K, Rhee P, Kulavatunyou N, Haider A, Phelan HA, et al. Emergency general surgery specific frailty index: A validation study. The journal of trauma and acute care surgery. 2016;81(2):254-60.
9. Dowswell G, Bartlett DC, Futaba K, Whisker L, Pinkney TD. How to set up and manage a trainee-led research collaborative. BMC Med Educ. 2014;14:94.
10. Jelley B, Long S, Butler J, Hewitt J. Cohort profile: the Welsh Geriatric Registrar-Led Research Network (WeGeN): rationale, design and description. BMJ Open. 2017;7(2):e013031.
11. Charlson ME, Pompei P, Ales KL, MacKenzie CR. A new method of classifying prognostic comorbidity in longitudinal studies: development and validation. J Chronic Dis. 1987;40(5):373-83.
12. Rockwood K, Song X, MacKnight C, Bergman H, Hogan DB, McDowell I, et al. A global clinical measure of fitness and frailty in elderly people. CMAJ : Canadian Medical Association journal = journal de l'Association medicale canadienne. 2005;173(5):489-95.
13. Copeland GP, Jones D, Walters M. POSSUM: a scoring system for surgical audit. Br J Surg. 1991;78(3):355-60.
14. RD Dripps. New classification of physical status. Anesthesio. 1963(24):1.

For peer review only

Appendix A: Case Report Form

Q1	Study ID	
Q2	Age at admission to study (years)	
Q3	Sex	Male Female
Q4	Comorbidities	CCF Y/N COPD Y/N<br>CVA Y/N Dementia Y/N<br>Hemiplegia Y/N CKD Y/N<br>Leukaemia Y/N DM(complicated) Y/N<br>Lymphoma Y/N DM(uncomplicated) Y/N Mild liver disease Y/N IHD Y/N<br>Severe liver disease Y/N PVD Y/N<br>Solid tumour Y/N Metastatic tumour Y/N<br>AIDS Y/N Peptic ulcer disease Y/N<br>Connective tissue disease Y/N<br>Other:______________________
Q5	Polypharmacy (≥5 medications)	Yes No
Q6	Care level prior to admission*<br><br>**The term "carer" to include both formal and informal care arrangements i.e. friends/*	Home (No carers*)<br>Home (with carers* ____ times/day)<br>Residential Home<br>Nursing home

	relatives	Intermediate care<br><br>Other: ______________
Q7	Frailty score	
Q8	Interval between admission & surgery (days)	
Q9a	Pre-operative ASA grade	
Q9b	Pre-operative PPOSSUM	Morbidity: Mortality:
Q10a	Operative indication	Peritonitis Perforation<br>Abdominal abscess Anastomotic leak<br>Intestinal fistula Sepsis (other)<br>Intestinal obstruction Haemorrhage<br>Ischaemia Colitis<br>Abdominal wound dehiscence<br>Abdominal compartment syndrome<br>Planned relook<br>Other ______________
Q10b	Procedure (circle **ALL** that apply)	Peptic ulcer (suture or repair of perforation)<br>Peptic ulcer (oversew of bleeding)<br>Gastric surgery - other<br>Small bowel resection<br>Colectomy: Left (including anterior resection)<br>Right Subtotal<br>Hartmann's procedure

		Colorectal resection – other<br>Haemostasis Enterotomy<br>Stoma formation Stoma revision<br>Adhesiolysis Intestinal bypass<br>Reduction of volvulus Washout only<br>Abdominal wall closure<br>Drainage of abscess/collection<br>Laparostomy formation<br>Repair of intestinal perforation<br>Resection of other intra-abdominal tumours<br>Exploratory/ re-look laparotomy only<br>Not amenable to surgery<br>Other ______________________
Q10c	Primary procedure type	Open<br>Laparoscopic<br>Laparoscopic converted to open<br>Laparoscopic-assisted
Q11a	Length of stay (days)	
Q11b	Readmission to hospital within 30 days	Yes No
Q11c	Reason for readmission	
Q12a	Post-operative complication within 30 days	Yes No
Q12b	Grade of complication	

Q13	Care level on discharge<br>**The term "carer" to include both formal and informal care arrangements i.e. friends/ relatives*	Home (No carers*)<br>Home (with carers* ____ times/day)<br>Residential Home<br>Nursing home<br>Other: ____________________
Q14	90 day mortality	Yes No
Q15	Length of ICU/HDU stay	ICU HDU<br>Days total stay __________
Q16	Intermediate care stay (days)	Yes No<br>Days total stay __________

Appendix B: Canadian Study of Health and Ageing (CSHA) Frailty Score (Rockwood Score)

The CSHA Frailty Scale	
**1 – Very fit**	Robust, active, energetic, well-motivated and fit; these people commonly exercise regularly and are in the most fit group for their age.
**2 – Well**	Without active disease, but less fit than people in category 1.
**3 – Well, with treated comorbid disease**	Disease symptoms are well controlled compared with those in category 4.
**4 – Apparently vulnerable**	Although not frankly dependent, these people commonly complain of being "slowed up" or have disease symptoms.
**5 – Mildly frail**	With limited dependence on others for instrumental* activities of daily living.
**6 – Moderately frail**	Help is needed with both instrumental* and non-instrumental activities of daily living.
**7 – Severely frail**	Completely dependent on others for activities of daily living, or terminally ill.

- *Non-instrumental activities of daily living are basic everyday tasks such as walking, bathing, dressing, toileting, brushing teeth and eating.*

For peer review only - http://bmjopen.bmj.com/site/about/guidelines.xhtml

Instrumental activities of daily living are further tasks such as cooking, shopping, driving etc. Further explanation is available at the following link if required:

https://asourparentsage.net/2009/12/17/adls-and-iadls-whats-the-difference/

Appendix C:

Clavien-Dindo Classification of Surgical Complications

Clavien-Dindo Classification of Surgical Complications	
Grade I	Any deviation from the normal postoperative course without the need for pharmacological treatment or surgical, endoscopic, and radiological interventions. Allowed therapeutic regimens are: drugs such as antiemetics, antipyretics, analgetics, diuretics, electrolytes, and physiotherapy. This grade also includes wound infections opened at the bedside.
Grade II	Requiring pharmacological treatment with drugs other than allowed for grade I complications. Blood transfusions and total parenteral nutrition are also included.
Grade IIIa	Surgical, endoscopic, or radiological intervention that is not under general anesthesia
Grade IIIb	Surgical, endoscopic, or radiological intervention that is under general anesthesia
Grade IVa	Life-threatening complication requiring intermediate care or intensive care unit management, single organ dysfunction (including dialysis, brain hemorrhage, ischemic stroke, and subarrachnoidal bleeding)
Grade IVb	Life-threatening complication requiring intermediate care or intensive care unit management, multi-organ dysfunction (including dialysis)
Grade V	Death of a patient
Suffix "d"	If the patient suffers from a complication at the time of discharge, the suffix "d" (for "disability") is added to the respective grade of complication. This label indicates the need for a follow-up to fully evaluate the complication

Appendix D: Definitions

Day of study – this is defined by the number of 24h periods passed from first attendance at hospital, the event being examined (e.g. date of operation). Patients admitted and the event occurs with the first 24hrs are classed as 1 day.

Q1. Combination of centre Number and record number. For example if you are in centre 024 and recording data on the 35th patient, the number would be 024.35

Q2. Age in completed years on date of admission to hospital

Q3. Please indicate sex of patient

Q4. These are comorbidities as defined by the Charlson Comorbidity Index. Each should be marked as present if there is any previous documented history of each diagnosis.

**Myocardial infarct**	History of medically documented myocardial infarction
**Congestive heart failure**	Symptomatic congestive heart failure w/ response to specific treatment
**Peripheral vascular disease**	Intermittent claudication, peripheral arterial bypass for insufficiency, gangrene, acute arterial insufficiency, untreated aneurysm (≥6cm)
**Cerebrovascular disease (except hemiplegia)**	History of TIA, or CVA with no or minor sequelae
**Dementia**	Chronic cognitive deficit
**Chronic pulmonary disease**	Symptomatic dyspnoea due to chronic respiratory conditions (inc. asthma)
**Connective tissue**	SLE, polymyositis, polymyalgia rheumatic, moderate to severe

**disease**	rheumatoid arthritis
**Peptic ulcer disease**	Patients who have required treatment for peptic ulcer disease
**Mild liver disease**	Cirrhosis without portal hypertension, chronic hepatitis
**Diabetes (without complication)**	Diabetes with medication
**Diabetes with end organ damage**	Retinopathy, neuropathy, nephropathy
**Hemiplegia (or paraplegia)**	Hemiplegia or paraplegia
**Moderate of severe renal disease**	Creatinine>265umol/L, dialysis, transplantation, uraemic syndrome
**Solid tumour (non-metastatic)**	Initially treated in the last 5 years exclude non-melanomatous skin cancers and in situ cervical carcinoma
**Leukaemia**	CML, CLL, AML, ALL, PV
**Lymphoma, Multiple myeloma**	Non-Hodgkin's Lymphoma, Hodgkin's, Waldenstrȍm, multiple myeloma
**Moderate or severe liver disease**	Cirrhosis with portal hypertension +/- variceal bleeding
**Metastatic solid tumour**	Metastatic solid tumour
**AIDS**	AIDS & AIDS-related complex

For peer review only - http://bmjopen.bmj.com/site/about/guidelines.xhtml

Q5. Polypharmacy counted as five or more prescribed regular medications on admission. This includes regular eye drops, inhalers and analgesia.

Q6. Care level prior to admission. Classed as level of social care input prior to admission. Please indicate only one. If patient at own home with daily care input please indicate the number of times each day carers attend.

Q7. Frailty score, 1-7 using the modified Rockwood Scale (please see Appendix B)

Q8. Interval between admission and emergency procedure. Classed as whole days, rounded up to the nearest whole day. (e.g. 0-24h classed as 1 day, 24-48h classed as 2 days)

Q9a. American Society of Anaesthesiologist (ASA) grade:

Grade	Description
1	Healthy individual with no systemic disease
2	Mild systemic disease not limiting activity
3	Severe systemic disease that limits activity but is not incapacitating
4	Incapacitating systemic disease which is constantly life-threatening
5	Moribund, not expected to survive 24 hours with or without surgery

Q9b. P-POSSUM Score: Calculated from pre-morbid status using multiple markers of baseline function including age, cardiac status, observations and blood test results. This should already be routinely documented in all patient notes as part of the National Emergency Laparotomy Audit dataset. If required please use the calculator found at http://www.riskprediction.org.uk/index-pp.php

Q10a. Primary operative indication as per National Emergency Laparotomy Audit data collection

Q10b. Primary operative procedure as per National Emergency Laparotomy Audit collection tool

Q10c. Primary surgical method used during the procedure. NOTE: Laparoscopic assisted should be used if decision to proceed to open was part of the pre-operative procedure planning, otherwise laparoscopic converted to open should be used.

Q11a. Total length of stay of primary admission **is** defined as number of 24h periods or part thereof, passed from first attendance at hospital, to discharge. Patients admitted and discharged with the first 24hrs are not included in the study, between 24-48hrs 2 day etc.

Q11b. Readmission to hospital within 30 days as an emergency (classed as whole days, rounded up to the nearest whole day) regardless of cause.

Q11c. Reason for readmission to hospital

Q12a. Post-operative complications include:

**Abdominal wall dehiscence**	Full thickness dehiscence of laparotomy wound within 30 days of discharge

**Anastomotic leakage**	A clinical diagnosis will require symptoms related to leakage (gas, pus, or faecal discharge from the drain site, peritonitis or discharge of pus from the rectum). In the event of a clinically suspicious leak (fever or abdominal pain) the diagnosis can be established by operative or radiological diagnosis. When an anastomosis is defunctioned the presence or absence of a leak will be established by contrast radiology.
**Urinary tract infection**	Patient needs to meet two of the following criteria:<br>• Fever >38^{0}C<br>• Suprapubic tenderness<br>• Costovertebral angle pain or tenderness<br>• Urinary urgency<br>• Urinary frequency<br>• Dysuria<br>• Urine culture with no more than two species of organisms identified, at least one of which is a bacterium of ≥105 CFU/mL
**Pneumonia**	Patient must meet one of the following criteria:<br>• Dullness to percussion on physical examination of chest and any of the following:<br>- New onset of purulent sputum or change in character of sputum<br>- Organism isolated from blood culture<br>- Isolation of pathogen from specimen obtained from transtracheal aspirate, bronchial brushing or biopsy

	• Chest radiographic examination shows new or progressive infiltrate, consolidation, cavitation or pleural effusion and any of the following<br>- New onset of purulent sputum or change in character of sputum<br>- Organism isolated from blood culture<br>- Isolation of pathogen from specimen obtained from transtracheal aspirate, bronchial brushing or biopsy<br>- Isolation of virus or detection of viral antigen in respiratory secretions<br>- Diagnostic single antibody titre (IgM) or four-fold increase in paired serum samples (IgG) for pathogen
**Superficial SSI**	Patient must meet one of these criteria<br>• Purulent drainage from the incision<br>• At least two of – pain, localised swelling, redness, head, fever AND the incision is opened deliberately to manage infection or the clinician diagnoses a SSI<br>• Wound organisms AND pus cells from aspirate/swab
**Deep (intra-abdominal) SSI**	Patient must meet one of these criteria<br>• A clinical diagnosis of wound infection with dehiscence of mass closure or any layer below fat/scarpa's fascia<br>• A clinical diagnosis of intra-abdominal collection

	(fever/abdominal pain) with operative/radiological evidence of a collection
**Cardiac**	All complications newly diagnosed within 30 days of discharge (e.g. AF, MI, etc.), even if unrelated to primary admission
**DVT/PE**	Radiologically confirmed within 30 days of discharge
**Radiological drain**	Any additional procedure after operation, including image guided aspiration of collection or placement of a drain.
**Reoperation**	Any return to theatre for a general surgical cause within 30 days of discharge
**Unplanned HDU/ITU admission**	Any unplanned episodes, even if unrelated to primary presentation

Q12b. Classification of complication using Clavien-Dindo Classification. Graded 1-5 – see Appendix C.

Q13. Care level on discharge. Classed as level of social care input after admission. Please indicate <u>only one</u>. If patient at own home with daily care input please indicate the number of times each day carers attend. If patient is discharged to intermediate care then please record the place of discharge from intermediate care.

Q14. 90 day mortality – counted in whole days, rounded up to the nearest day

Q15. ICU/HDU stay – counted in whole days, rounded up to the nearest day

Q16. Intermediate care length of stay - counted in whole days, rounded up to the nearest day
